# Acknowledgement of manuscript reviewers 2017

**DOI:** 10.18332/ejm/83702

**Published:** 2018-01-22

**Authors:** G Victoria

**Affiliations:** 1 Department of Midwifery, Technological Educational Institute of Athens, Greece

## CONTRIBUTING REVIEWERS

The editors of ***European Journal of Midwifery*** would like to thank all our reviewers who have contributed to the journal in Volume 1 (2017).

**Carrie Bonsack**

United States

**Signe Egenberg**

Norway

**Ramón Escuriet**

Spain

**Jennifer Fenwick**

Austria

**Belén Ferrer**

Spain

**Susan Fleming**

United States

**Peter Gallagher**

New Zealand

**Jane Henderson**

United Kingdom

**Bengt Kayser**

Switzerland

**Julia Leinweber**

Australia

**Colin Martin**

United Kingdom

**Rui Filipe Oliveira Miguelote**

Portugal

**Polona Mivšek**

Slovenia

**Jane Morrow**

Australia

**Jaana Nevalainen**

Finland

**Rhona O’Connell**

Ireland

**Victor Pulgar**

United States

**Mirjam Snellen**

Australia

**Chuenjitwongsa Supachai**

Thailand

**Linda Sweet**

United States

**Stefania Triunfo**

Spain

